# Uncertainty modulates visual maps during noninstrumental information demand

**DOI:** 10.1038/s41467-022-33585-2

**Published:** 2022-10-07

**Authors:** Yvonne Li, Nabil Daddaoua, Mattias Horan, Nicholas C. Foley, Jacqueline Gottlieb

**Affiliations:** 1 Department of Neuroscience, Columbia University, New York, NY USA; 2 Mortimer B. Zuckerman Mind Brain Behavior Institute, Columbia University, New York, NY USA; 3 Kavli Institute for Brain Science, Columbia University, New York, NY USA

**Keywords:** Neuroscience, Medical research

## Abstract

Animals are intrinsically motivated to obtain information independently of instrumental incentives. This motivation depends on two factors: a desire to resolve uncertainty by gathering accurate information and a desire to obtain positively-valenced observations, which predict favorable rather than unfavorable outcomes. To understand the neural mechanisms, we recorded parietal cortical activity implicated in prioritizing stimuli for spatial attention and gaze, in a task in which monkeys were free (but not trained) to obtain information about probabilistic non-contingent rewards. We show that valence and uncertainty independently modulated parietal neuronal activity, and uncertainty but not reward-related enhancement consistently correlated with behavioral sensitivity. The findings suggest uncertainty-driven and valence-driven information demand depend on partially distinct pathways, with the former being consistently related to parietal responses and the latter depending on additional mechanisms implemented in downstream structures.

## Abstract (web summary)

Curiosity is motivated by uncertainty and valence, but how uncertainty and valence are encoded in the brain remains poorly understood. Here, the authors show that parietal neurons are enhanced by both factors, but that they specifically predict visual information seeking based on uncertainty.

## Introduction

Humans and other animals have an intrinsic desire to obtain information about future events. This desire is clearly illustrated in tasks of noninstrumental information demand in which participants have the opportunity to obtain information about future non-contingent rewards but cannot take actions to control the reward. Animals, including humans and monkeys, are willing to pay and exert effort to obtain noninstrumental information, showing that they value information as a good in itself, independently of any material gains they may realize by acting on the information^1,2^.

Studies of noninstrumental information demand suggest that the intrinsic utility of gathering information is of two kinds. On the one hand, participants seek to resolve uncertainty by gathering accurate information^1,3–6^. This is an important mechanism through which individuals can improve their predictions in an unbiased, theoretically normative fashion. On the other hand, participants prefer to obtain observations that have positive rather than negative valence—e.g., signal the availability of reward rather than a lack of reward^3,5,7^. This preference is thought to reflect an emotional bias, or “anticipatory utility”, whereby individuals seek to anticipate (“savor”) desirable outcomes but avoid anticipating (“dreading”) undesirable outcomes^8,9^. Anticipatory utility can produce non-normative biases whereby people reject information that is likely to signal undesirable outcomes, but nevertheless, it persists even in instrumental conditions when it limits the ability to take appropriate actions^5,10,11^ (e.g., people may avoid information about a medical diagnosis). Moreover, the relative strengths of anticipatory utility and uncertainty motives show individual variability that correlates with personality traits^12,11^.

Despite the importance of different information demand strategies, their neural mechanisms are not well understood. An imaging study in humans suggests that uncertainty resolution and anticipatory utility are mediated by different structures involved in reward and motivation^7^. However, humans and monkeys often obtain information through active sensing behaviors like making rapid eye movements (saccades) to visual stimuli, but little is known about how motivational signals of reward and uncertainty interface with visuo-motor areas that generate saccade policies.

Saccades are controlled by a network of topographically organized cortical and subcortical areas in which neurons have visual receptive fields (RF) and encode saccade goals. The lateral intraparietal area (LIP) is an intermediate node in this network that encodes “visual priority”—a sparse topographic representation of relevant stimuli that provides inputs to downstream motor mechanisms deciding how to act on the stimuli^13,14^. In contrast, reward probability and uncertainty are encoded outside the visual system^15–17^, including in a network involving the pallidum, dorsal striatum, and dorsal anterior cingulate cortex (dACC)^18^. Uncertainty-sensitive cells in this network are not spatially tuned and cannot specify a saccade target location, raising the question of how motivational and uncertainty signals shape concrete saccade policies.

Here we examined this question by recording LIP cells in a task of noninstrumental information demand, in which monkeys were free (but not trained) to reveal informative stimuli in trials with different reward probability. To dissociate the effects of valence and uncertainty, we fit the data with two functions of reward probability^7,19^. One function increased linearly with reward probability and measured the impact of valence—the extent to which the demand for information depended on the probability that the information would signal a desirable outcome. The second function was a quadratic (inverted-U) function of reward probability that peaked at 50% and was minimal at 0 and 100% reward probability and measured the sensitivity to uncertainty—the extent to which information demand at an intermediate (uncertain) probability departed from the predictions of a linear trend. As in previous studies using this method^7,18–20^, we verified that reward and uncertainty regressors had low correlations in our dataset, establishing their suitability to disambiguate the effects of valence and uncertainty.

We show that monkeys were sensitive to both valence and uncertainty and, consistent with previous findings in humans, showed individual differences in the relative strengths of these motives^7,12,19^. Importantly, these individual differences were not encoded in LIP, suggesting that the parietal cortex is more closely associated with uncertainty-based relative to valence-based information gathering.

## Results

### Information seeking is sensitive to reward probability and uncertainty

**Fig. 1:**
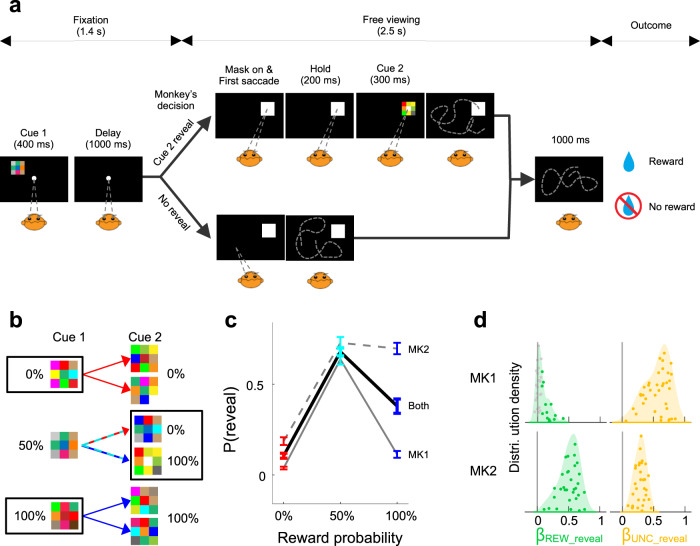
Task and behavior. **a** Trial structure in the information-seeking task. The monkeys started with a period of central fixation in which they viewed Cue 1 followed by a delay period. Gaze was then released for 2.5 s of free-viewing. During the first 1.5 s of free-viewing, the monkeys could hold gaze on the mask if they wished to reveal Cue 2. After an additional 1 s delay the outcome (reward or lack of reward) arrived regardless of free-viewing behavior. **b** Cue-reward contingencies. Cue 1 and Cue 2 were distinct checkerboards associated with reward probabilities whose informativeness was inversely related. When Cue 1 signaled 0 or 100% reward probabilities it provided complete information about the trial’s outcome (black frames) and Cue 2 was merely redundant. When Cue 1 signaled 50% probability, it provided no new information, and all the information was provided by Cue 2 (black frame). Each Cue 1 pattern was equally likely to be followed by one of two Cue 2 patterns, controlling for visual novelty. **c** Reveal probability. P(reveal) was the fraction of trials where the monkeys revealed Cue 2 (mean and SEM over 37 sessions in MK1 and 31 sessions in MK2). Here and in all following figures, red, cyan, and blue represent, respectively, 0, 50, and 100% reward probability. **d** Distributions of the coefficients indicating the effects on revealing behavior of reward probability (*β*_*REW_reveal*_, green) and uncertainty (*β*_*UNC_reveal*_, yellow). Each point is one session. Colored points indicate regression coefficients that are significantly different from 0 (*p* < 0.05). The shading represents probability density, with points randomly jittered along the *y*-axis within the distribution envelope for visualization. Source data are provided as a Source Data file.

Two monkeys performed a task in which they were free to obtain advanced information about noncontingent rewards (Fig. 1a). A trial started with central fixation, followed by the presentation of a peripheral cue (Cue 1) that signaled the trial’s reward probability. Cue 1 was followed by a delay period in which the monkeys maintained central fixation and a free-viewing period in which the monkeys could reveal an additional reward cue (Cue 2). During the first part of free-viewing, the monkeys had access to a visual mask which, contingent on the monkeys’ maintaining gaze on the mask, disappeared and revealed Cue 2. After a fixed interval of 2.5 s, the trial ended with the delivery of the outcome—a reward or a lack of reward. The outcome was noncontingent on the monkeys’ free-viewing behavior, which thus expressed the monkeys’ intrinsic willingness to reveal Cue 2.

To determine how this willingness depended on valence and uncertainty, we analyzed behavior using two functions of reward probability (Fig. 1b). The valence that Cue 2 was expected to have (the probability that it would convey a positive outcome) increased linearly with reward probability. In contrast, the uncertainty that Cue 2 would resolve was a nonlinear function of reward probability, being low at 0 and 100% but maximal at 50%. Thus, Cue 1 sets up the valence and uncertainty associated with sampling Cue 2. Moreover, the informativeness of the two stimuli was inversely related. When Cue 1 was fully informative (signaling 0 or 100% reward probability), it rendered Cue 2 uninformative, and when Cue 1 was uninformative (signaling a 50% probability), it rendered Cue 2 maximally informative (frames in Fig. 1b).

The monkeys’ information demand was sensitive to both factors (Fig. 1c). The willingness to reveal Cue 2 was higher for 100% than for 0% trials, suggesting sensitivity to anticipatory utility. Moreover, the willingness to reveal in 50% trials was higher than would be predicted by a linear trend, suggesting an effect of uncertainty. We quantitatively measured each factor by fitting viewing behavior with a two-parameter regression in which one term linearly coded for reward probability (**REW**, 0.0, 0.5, and 1.0) and a second term indexed uncertainty (**UNC**, respectively, 0, 1, 0; Methods, Eq. 1). The coefficients for the linear term were on average positive, confirming a significant valence effect in each monkey (*β*_*REW_reveal*_ relative to 0: MK1: *p* < 10^−4^, MK2, *p* < 10^−5^; Wilcoxon test relative to 0). Likewise, both monkeys were strongly sensitive to uncertainty, with the uncertainty coefficients being significantly greater than 0 on average (Fig. 1d, yellow; *β*_*UNC_reveal*_ relative to 0, MK1: *p* < 10^−6^, MK2, *p* < 10^−5^) and, remarkably, in every individual session (Fig. 1d, colored points). The Spearman correlation between the **REW** and **UNC** regressors was below 0.07 in all individual sessions (all *p* > 0.1), showing that the model was suitable for disentangling the effects of these factors. Moreover, because regressors compete to explain variance in a multiple regression model, finding significant coefficients indicated that reward and uncertainty each explained behavior beyond the effect captured by the other term. A separate analysis of the fraction of unique variance explained based on R^2^ (Methods) replicated all the effects based on the coefficients (Supplementary Fig. 3), further confirming that reward and uncertainty explained unique fractions of behavioral variability.

While both monkeys had significant sensitivity to anticipatory utility and uncertainty, the relative strengths of these drives had individual variability. The effect of reward was stronger in MK2 relative to MK1, as shown by his higher *β*_*REW*_ coefficients (Fig. 1d, green; *p* < 10^−10^ between monkeys, Wilcoxon test) and a higher fraction of sessions with significant effects (100 vs 37%). Conversely, the effect of uncertainty was stronger in MK1 relative to MK2, as shown by MK1’s higher *β*_*UNC*_ coefficients (Fig. 1d, yellow; *p* < 10^−6^, Wilcoxon test). These differences could be explained neither by the animals’ training (which involved identical protocols; Methods) nor by their familiarity with the cues (since both monkeys had anticipatory licking responses that closely followed the cued reward probabilities; Supplementary Fig. 1a) or by their engagement with the task (since both monkeys showed significant effects of valence and uncertainty). Thus, the findings reflect individual differences in the monkeys’ relative sensitivities to uncertainty and anticipatory utility, consistent with previous studies on humans^12^. We return to this finding below in relation to neural activity.

Control analyses ruled out spurious explanations for the monkeys’ information-seeking behavior. Anticipatory licking was influenced by reward probability even when the monkeys did not reveal Cue 2, showing that the monkeys understood that rewards were forthcoming regardless of revealing behavior (Supplementary Fig. 1b). Viewing behavior was unchanged in control sessions in which the spout was placed inside the monkeys’ mouth, ruling out that the monkeys revealed Cue 2 to reduce the physical effort of licking^19^. Finally, each Cue 1 was equally likely to be followed by one of two Cue 2 patterns (Fig. 1b), ruling out that revealing behavior was related to differential expectations of visual novelty.

### LIP neurons are modulated by reward and uncertainty

To examine the neural substrate of the sensitivity to reward and uncertainty, we recorded the activity of 68 LIP neurons (37 in MK1) that were selected based on their good isolation and spatial tuning—the presence of a circumscribed visual receptive field (RF) in a memory-guided saccade task (Supplementary Fig. 2a, b). For each neuron, the stimuli appeared inside the RF or at two possible locations outside the RF (Methods). The locations of Cue 1 and the mask were independently randomized with the constraint that they did not overlap in an individual trial.

**Fig. 2:**
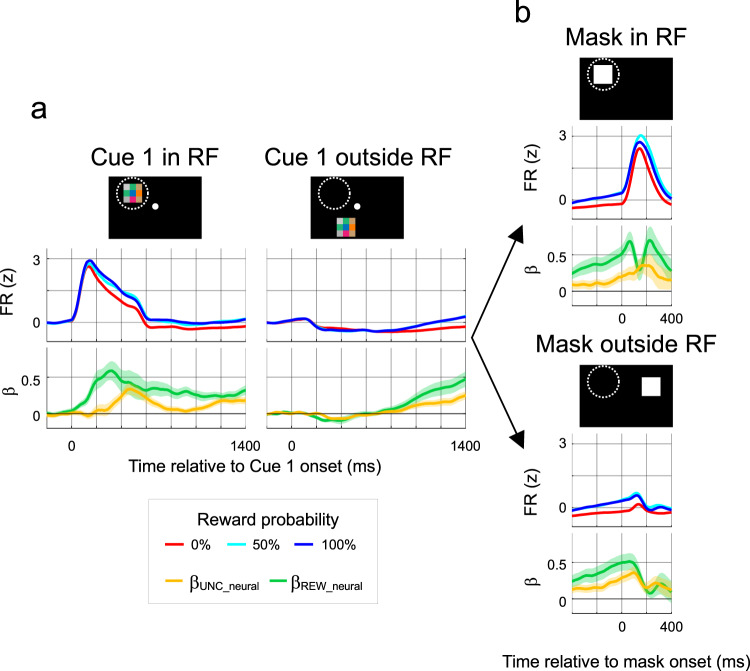
LIP modulations by reward and uncertainty. **a** Responses aligned to Cue 1 onset. The cartoons show Cue 1 (colored checkerboard) appearing inside or outside the RF (dashed circle). The middle row shows population peri-stimulus response histograms computed by z-scoring the raw FR within each neuron (using all of its data in the task), averaging across trials within each neuron, and averaging across neurons (*n* = 68). The bottom panels show regression coefficients indicating the effects of uncertainty (*β*_*UNC_neural*_, yellow) and reward (*β*_*REW_neural*,_ green). The traces are averaged across cells (*n* = 68) and shading shows ±2 SEM (equivalent to 95% confidence interval). **b** Responses aligned to mask onset. Same format as in **a**. Saccades away from fixation had an average latency of 193.2 ± 0.22 ms. Across the trials contributing to the analyses in the different panels, the Spearman correlations between **REW** and **UNC** regressors were not significant for any cell (range, −0.11 to 0.19). Source data are provided as a Source Data file.

We first focused on responses to Cue 1 during central fixation, when the retinotopic locations of Cue 1 and the mask were controlled and could be inside or outside the visual receptive field (RF). As expected from their visuo-spatial selectivity, the neurons had excitatory responses if Cue 1 appeared inside the RF (Fig. 2a, “Cue 1 in RF”) but not when it appeared outside the RF (Fig. 2a, “Cue 1 outside RF”). Similarly, the cells responded to mask onset if the mask appeared inside the RF but not outside the RF (Fig. 2b, “Mask in RF” vs “Mask outside RF”). At all stimulus geometries, these responses were modulated by reward and uncertainty. Firing rates were higher for 100% than 0% probability and, for 50% probability were higher than would be predicted by a linear trend (Fig. 2).

To determine if these responses indicated distinct sensitivity to reward and uncertainty, we fit individual neurons with a model that included the **REW** and **UNC** terms we used for behavior, alongside nuisance regressors to control for other factors previously proposed to be encoded by the cells (the direction and latency of the first saccade away from fixation, licking behavior, and the prior trial reward^21^; Methods, Eq. 3). This revealed significant effects of reward probability and uncertainty at each stimulus geometry. After onset of Cue 1 inside the RF, the neural coefficients *β*_*UNC_neural*_ and *β*_*REW_neural*_ were significantly positive, indicating that reward and uncertainty enhanced firing rates throughout the visual epoch (100–600 ms: *β*_*UNC_neural*_: 0.16 ± 0.03, *p* < 10^−6^, *β*_*REW_neural*_ 0.41 ± 0.06, *p* < 10^−9^) and delay period (1000–1400 ms; *β*_*UNC_neural*_: 0.14 ± 0.02, *p* < 10^−9^, *β*_*REW_neural*_ 0.25 ± 0.02, *p* < 10^−11^, Wilcoxon test relative to 0, *n* = 68). Significant reward and uncertainty sensitivity were also found in the visual response to the mask (Fig. 2b, Mask in RF; 0–400 ms; *β*_*UNC_neural*_: 0.25 ± 0.04, *p* < 10^−6^; *β*_*REW_neural*:_ 0.51 ± 0.05, *p* < 10^−10^, *n* = 68; see also Methods). Moreover, reward and uncertainty modulations were robust even when no stimulus appeared inside the RF—after Cue 1 presentation (Fig. 2a, right; 1000–1400 ms, *β*_*UNC_neural*_: 0.16 ± 0.02, *p* < 10^−9^; *β*_*REW_neural*_: 0.34 ± 0.04, *p* < 10^−9^, *n* = 68) or after onset of the mask outside the RF (Fig. 2b, bottom; *β*_*UNC_neural*_*:* 0.23 ± 0.03, *p* < 10^−8^; *β*_*REW_neural*_: 0.27 ± 0.03, *p* < 10^−8^, *n* = 68). Except for higher *β*_*REW*_ coefficients after mask onset inside versus outside the RF (*β*_*REW_neural*:_ *p* < 10^−6^), coefficients had comparable magnitudes across stimulus geometries (paired tests in vs out RF, *n* = 68, Cue 1, delay: *β*_*UNC_neural*_, *p* = 0.33, *β*_*REW*_, *p* = 0.12; mask: *β*_*UNC_neural*_: *p* = 0.23). As expected, when Cue 1 appeared in the RF, some neurons were sensitive to the visual patterns (*Methods*), but the presence of reward and uncertainty modulations were robust at all stimulus geometries ruling out explanations in terms of pattern sensitivity.

As in the behavioral data analysis, it was important to verify that reward and uncertainty explained distinct portions of firing rate variability. In the sets of trials contributing to each of the above analyses, reward and uncertainty regressors had very low correlations (ranging between −0.11 to 0.19 across cells, all *p* > 0.1), meaning that the effects could be reliably separated with regression analysis. Moreover, analyses of unique variance explained (R^2^, *Methods*) confirmed the results of the coefficient analyses and showed that they were robust in individual monkeys (Supplementary Fig. 3a, b). At times of peak modulation, reward and uncertainty explained, respectively, ~10 vs ~5% of unique firing rate variability in the population response with substantial fractions of individual cells showing significant contributions from each factor (e.g., after the onset of the mask in RF, over 40% of cells were sensitive to uncertainty and over 80% were sensitive to reward in each monkey, and similar results were found in other task epochs and geometries; see Supplementary Fig. 3c, d). Thus, reward and uncertainty explained independent fractions of firing rate variance in each individual monkey.

### Uncertainty modulations are independent of saccade plans

**Fig. 3:**
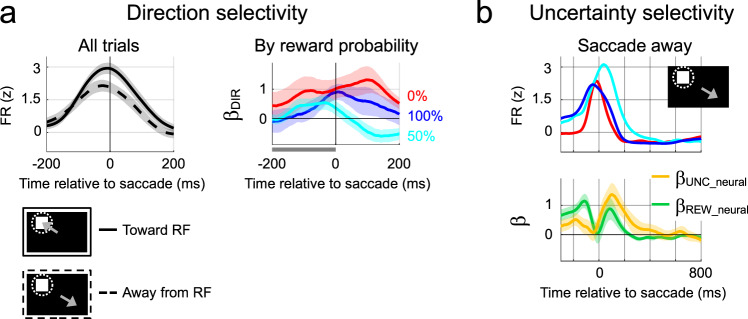
Uncertainty and saccade direction. **a** Directional selectivity. (Left) PSTH of activity aligned on saccade onset, pooled across reward probabilities for the geometry depicted in the cartoons (mask inside the RF and saccades toward (solid) and away (dashed) from the RF). The traces show mean and ±2 SEM for all cells with sufficient trials in each condition (saccade away, *n* = 68; saccade toward, *n* = 52). (Right) Directional selectivity by reward probability. Time-resolved regression coefficients capturing the effect of saccade direction (*β*_*DIR*_) in the same trials as left panel separated by reward probability (mean and ±2 SEM across cells with sufficient trials; 0%; *n* = 31; 50%: *n* = 26; 100%: *n* = 21). Gray bar along the *x*-axis indicates time window used for statistics. **b** Effects of reward and uncertainty for saccades away from a mask in the RF (cartoon). PSTHs (top) and reward and uncertainty coefficients (bottom) aligned on saccade onset (mean and ±2 SEM, *n* = 26 cells with sufficient number of trials). Other conventions as in Fig. 2. The correlation of **UNC** and **REW** regressors ranged between −0.04 and 0.1, and was never significant. Source data are provided as a Source Data file.

By including saccade direction as a nuisance regressor, the analyses above established that the reward and uncertainty modulations were not artefacts of a saccade planning response. However, consistent with their spatially tuned activity (Supplementary Fig. 2a, b), the neurons had a slight saccadic response; if the mask was inside the RF, firing rates were slightly higher if the saccade was directed toward versus away from the mask (Fig. 3a, top), resulting in significant saccade direction coefficients in a brief period around the saccade (*β*_*DIR*_; Fig. 3a, top right).

The saccade directional coefficients were equivalent across reward probabilities, suggesting that reward and uncertainty modulations were independent of motor response (Fig. 3a, top right, pre-saccade time window; *p* = 0.087, one-way ANOVA; *n* = 31, 26, and 21 cells for, respectively, 0, 50, and 100% probability). To further verify this conclusion, we estimated the reward and uncertainty modulations in the subset of trials in which the mask appeared inside the RF, but the saccade was directed away and did not reveal Cue 2. As shown in Fig. 3b, *β*_*UNC_neural*_ and *β*_*REW_neural*_ coefficients were highly robust in these trials, starting before the saccade and extending for several hundreds of milliseconds after saccade onset (average coefficients in the 200 ms after saccade onset, *β*_*UNC_neural*_,1.06 ± 0.15, *p* < 10^−4^; *β*_*REW_neural*_ = 0.54 ± 0.14, *p* < 10^−3^, Wilcoxon test against 0, *n* = 26). This was confirmed by analysis of variance explained, which produced significant population-level R^2^ for reward (0.09 ± 0.014) and uncertainty (0.03 ± 0.007, both *p* < 10^−3^, Wilcoxon test against label-shuffled results) and in, respectively, 58 and 39% of individual cells (one-sided permutation tests, *p* < 0.05). These results are consistent with the view that LIP encodes a visual rather than motor priority map^22^. It suggests that the neurons encode the higher priority of stimuli that are associated with a positive valence or uncertainty resolution independently of an immediate saccade motor response.

### Neural uncertainty modulations predict behavioral sensitivity

Having found that the uncertainty modulations were independent of the immediate saccade plan, we wondered if these modulations may predict the monkeys’ sensitivity to uncertainty on longer time scales. We thus asked if the neural and behavioral *β*_*UNC*_ coefficients were correlated across days. Although this analysis entails comparisons across cells, it is validated by the fact that we targeted a constant population of cells (our recordings were clustered in a small area of less than ~2 mm^2^ on the cortical surface, and the neurons’ responses on the benchmark memory-guided saccade task showed no change across days; Supplementary Fig. 2c, d).

**Fig. 4:**
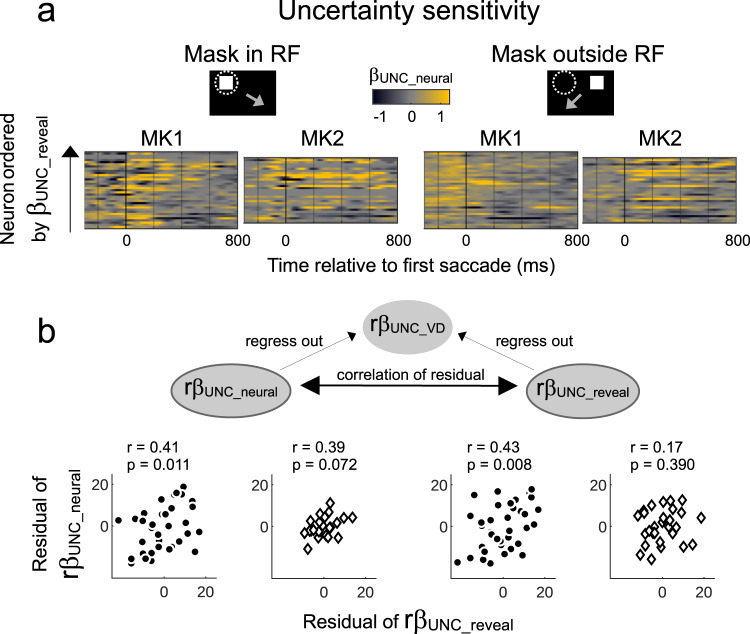
Correlations between neural and behavioral sensitivity to uncertainty. **a** Color maps of the time-resolved *β*_*UNC_neural*_ coefficients (color) for individual neurons ordered according to the magnitude of the uncertainty coefficient in revealing behavior (*β*_*UNC_reveal*_). The analysis was performed on trials when the mask was inside the RF (left) or outside the RF (right) but the saccade was away from the mask (cartoons). **b** Top: the partial correlation analysis controlling for correlations between the effects of uncertainty on viewing duration (VD) and reveal behavior. Raw *β*_*UNC_reveal*_ coefficients and *β*_*UNC_neural*_ averaged over 200-600 ms after saccade onset were transformed to ranked values (*rβ*) and separately regressed against the ranked uncertainty coefficients on VD (r*β*_*UNC_VD*_). The bottom scatterplots show the correlations between the residuals of these fits corresponding to the panels in **a**. Each point is one session, and shows the residual of r*β*_*UNC_neural*_ coefficient (ordinate) against the residual of r*β*_*UNC_reveal*_ coefficient (abscissa). The text shows the correlation coefficient and its p-value. In the subset of trials used for the analyses in **a** and **b**, the Spearman correlation coefficients between **UNC** and **REW** regressors ranged between −0.07 and 0.46 across cells. Source data are provided as a Source Data file.

Across this uniform neural population, the individual neuron *β*_*UNC*_ coefficients were significantly correlated with daily fluctuations in the sensitivity to uncertainty in the monkeys’ behavior. While the effects of reward and uncertainty were sustained, the neural-behavioral correlations were most consistent after mask onset and extended for several hundred milliseconds after this onset. We illustrate this result in Fig. 4a, by showing the individual neuron uncertainty coefficients as a function of time after mask onset and sorting the neurons in order of the revealed uncertainty coefficients. Uncertainty modulations were more pronounced and of longer duration in sessions with stronger behavioral modulation (more yellow toward the top of the maps). The correlations between *β*_*UNC_reveal*_ and *β*_*UNC_neural*_ coefficients (200–600 ms after mask onset) were significant if the mask appeared inside the RF in the combined dataset (Spearman rho = 0.46, *p* < 0.001, *n* = 59) and in individual monkeys (Fig. 4a, left; MK1, rho = 0.41, MK2, rho = 0.55, both *p* < 0.05). When the mask was outside the RF, the correlations reached significance in only one monkey (Fig. 4a, right; MK1: rho = 0.39, *p* < 0.05; MK2, rho = 0.30, *p* = 0.12). Note that, to rule out an effect of the immediate motor response, these analyses were restricted to trials in which the saccade was directed away from the mask; we found similar results when we included all-saccade trials, although these trials had higher variability related to the variability in the motor response and revealing Cue 2 (Supplementary Fig. 4).

Because the neural-behavioral correlations peaked relatively late after mask onset, we verified whether, rather than pre-saccadic selection, they reflected some aspect of post-saccadic behavior. The 200–600 ms epoch is the approximate time when the monkeys could have revealed and inspected the information shown by Cue 2. When the monkeys revealed Cue 2, their viewing durations (VD) were sensitive to valence and uncertainty^19^ (*β*_*UNC_VD*_; Methods, Eq. 2) and daily fluctuations of the uncertainty sensitivity in VD were correlated with those in revealing behavior (*β*_*UNC_VD*_ vs *β*_*UNC_reveal*_ across days: MK1, rho = 0.43, *p* < 0.01; MK2, rho = 0.39, *p* < 0.05), suggesting that the apparent neural-behavioral correlation may have arisen artefactually because of a relationship with VD. To examine this possibility, we conducted a nonparametric partial correlation analysis in which we examined the residuals after regressing out the effects of the (ranked) *β*_*UNC_VD*_ coefficients on the (ranked) *β*_*UNC_neural*_ and *β*_*UNC_reveal*_ coefficients (Methods and Fig. 4b, cartoon). The residual *β*_*UNC_neural*_ and *β*_*UNC_reveal*_ coefficients remained significantly correlated in each monkey (Fig. 4b, scatterplots), ruling out an artefactual effect of VD.

Together, the findings suggest that the uncertainty enhancement of LIP visual activity correlates with the effect of uncertainty on information gathering. The correlations persisted across recording sessions and cells, suggesting that the neural effects of uncertainty change on scales that are slower than an individual trial and are common to populations of LIP cells.

### Relationship between uncertainty and reward modulations

We next sought to examine the extent to which LIP neurons reflect the individual variability in the monkeys’ sensitivity to valence and uncertainty. As noted above, sampling behavior was relatively more sensitive to uncertainty in MK1 and more sensitive to reward in MK2 (Fig. 1c, d). Strikingly, these individual differences were not encoded by LIP cells, which showed stronger effects of reward relative to uncertainty in both monkeys, as noted above (Fig. 2 and Supplementary Fig. 3).

**Fig. 5:**
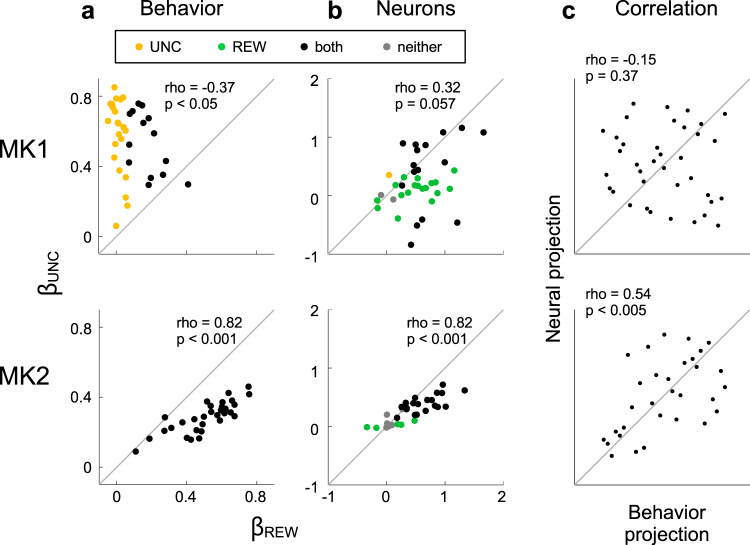
Relationship between uncertainty and reward modulations. **a** Scatter points show the correlation of *β*_*UNC_reveal*_ (ordinate) and *β*_*REW_reveal*_ (abscissa) coefficients for individual sessions, color coded to indicate significance (*p* < 0.05) of the coefficients. Significant uncertainty effects could appear alone (yellow) or in combination with reward sensitivity (black). The gray line is the unity line. Rho and *p*-value are of a Spearman correlation analysis. **b** Correlation of *β*_*UNC_neural*_ and *β*_*REW_neural*_ fit from average z-scored firing rates during the 400 ms after the mask appeared inside the RF of individual neurons. Conventions are the same as **a**. **c** Scatter points show the rank of PC projections (see text for details) of neural (ordinate) and behavior (abscissa) data. The gray line is the unity line. Rho and *p*-value are of a Spearman correlation analysis. Source data are provided as a Source Data file.

This analysis of the monkeys’ behavior also showed that the monkeys differed in the associations between their reward and uncertainty sensitivity. MK1 had a negative correlation between the *β*_*UNC_reveal*_ and *β*_*REW_reveal*_ coefficients (Spearman rho, respectively, −0.37, *p* = 0.025; Fig. 5a, top). As illustrated by the raw data in Supplementary Fig. 5, on days in which MK1 sampled more on 50% of trials, he tended to sample less on 100% of trials (Supplementary Fig. 5a, top). In contrast, MK2 had a positive correlation (Spearman rho, 0.82, *p* < 10^−6^, Fig. 5a, bottom). On days in which MK2 sampled more on 50% of trials, he also sampled more on 100% of trials (Supplementary Fig. 5a, bottom).

In contrast to this behavioral difference, the neural reward and uncertainty modulations showed only null or positive correlations in both monkeys. The visual response to the mask showed positive correlations between *β*_*UNC_neural*_ and *β*_*REW_neural*_ coefficients in both monkeys (Fig. 5b). Across the different task geometries, and whether we analyzed the coefficients or R^2^, we never found a negative correlation that would correspond to MK1’s behavioral pattern.

To analyze the relationship between uncertainty and reward sensitivity, we computed the principal components (PCs) linking the *β*_*UNC*_ and *β*_*REW*_ coefficients for behavior and neural activity (using the data in, respectively, Fig. 5a and b). The first PC identifies the main axis of common variability in the two metrics, and the projections of individual points onto this PC measure the extent to which reward and uncertainty sensitivity covaried in each session or cell. In MK2, the neural and behavioral projections were strongly correlated (Fig. 5c, bottom, Spearman rho = 0.54, *p* = 0.002), showing that, in sessions in which LIP neurons were more sensitive to both uncertainty and reward, the monkeys’ revealing behavior was also more sensitive to both factors. MK1, in contrast, showed no correlation (Fig. 5c, top, Spearman rho = −0.15, *p* = 0.375). For him, sessions in which LIP neurons were more sensitive to both uncertainty and reward did not result in greater behavioral sensitivity to these factors. Thus, the correlated variability in uncertainty and reward modulations in LIP cells can be significantly modified by downstream mechanisms producing the revealing behavior.

We finally asked if LIP neurons encoded the sensitivity to reward in revealing behavior. In MK2, the neural and behavioral reward coefficients were significantly correlated, as expected based on this monkey’s association of the two factors (Spearman rho for *β*_*REW_reveal*_ and *β*_*REW_neural*_ in the mask response, 0.5, *p* = 0.005; for the corresponding R^2^ values, 0.45, *p* = 0.01). However, this was not replicated in MK1, whose LIP reward modulations did not covary with his reward sensitivity (Spearman rho for *β*_*REW_reveal*_ and *β*_*REW_neural*_ (mask response), 0.22, *p* = 0.1857; for R^2^ values, 0.21, *p* = 0.2). Thus, LIP neurons show consistent correlations with the monkeys’ behavioral sensitivity to but not with their sensitivity to anticipatory utility.

### The neurons respond to the revealed visual information

**Fig. 6:**
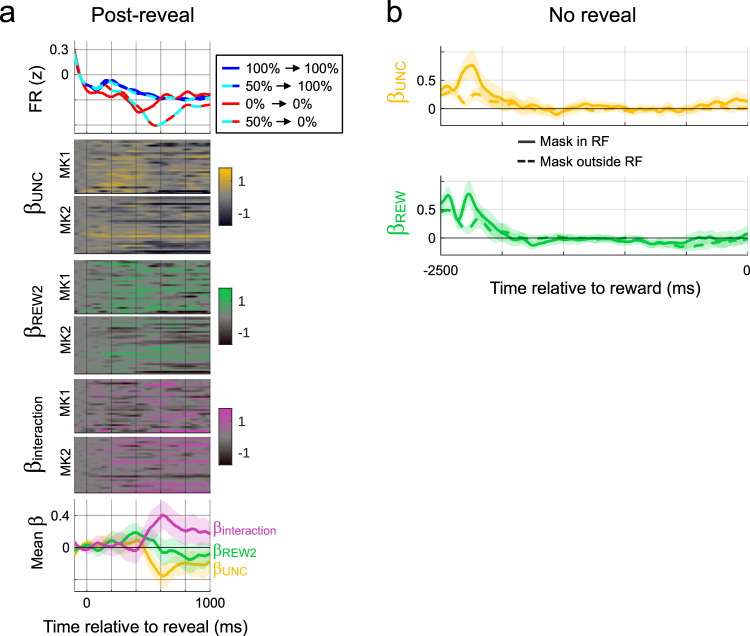
LIP neurons responded to new visual information. **a** Responses to the information conveyed by Cue 2. Top: Firing rates on trials in which Cue 2 was revealed, aligned on the time of reveal. PSTHs show average firing for the possible combinations of reward and uncertainty resolved by Cue 2 (*n* = 60 cells with at least 2 trials in each condition). Pre-reveal firing was subtracted to remove effects of Cue 1. Heatmaps show time-resolved regression coefficients (uncertainty, reward and interaction) for each neuron included in the top panel. The bottom panel shows the average coefficients (shading shows ±2 SEM). **b** Lack of anticipation of information on no-reveal trials. The panels show uncertainty and reward coefficients on no-reveal trials, aligned on the time of reward onset (mean and ±2 SEM, *n* = 59/68 cells for mask in/outside RF with at least 2 trials in each condition). Source data are provided as a Source Data file.

In the analyses conducted so far, we explored the effects of reward and uncertainty conveyed by Cue 1, but we wondered if the neurons may have additional independent responses to the information conveyed by Cue 2. This is an interesting question because Cue 2 was outside the RF (at the monkeys’ fixation), and thus a response to its information would be a global effect. To examine this question, we focused on trials in which the monkeys revealed Cue 2, and plotted neural activity aligned on reveal onset after subtracting the average pre-reveal firing rates to remove confounds related to Cue 1-based expectations (Fig. 6a).

This showed that, if Cue 2 announced a reward, firing rates showed no significant change (Fig. 6a, top, blue and blue/cyan traces). However, if Cue 2 announced a lack of reward, firing rates showed a prominent dip reaching the lowest level 600 ms after the reveal (red and red/cyan traces). The dip did not appear to be a reward prediction error response because it was present even on 0% trials when Cue 2 was redundant. However, the dip was more pronounced if Cue 2 resolved uncertainty rather than merely confirming prior expectations (Fig. 6a, top panel, cyan-red vs solid red traces), showing that it was enhanced by uncertainty. Thus, the neurons conveyed nonspatial responses to the reward conveyed by Cue 2. These responses were distinct from the Cue 1 evoked activity but nonetheless sensitive to the predictions that had been made by that cue.

We quantified these effects using a linear model with regressors indicating the reward signaled by Cue 2 (REW2, 0 or 1), the uncertainty resolved by Cue 2 (UNC, 0, 1,0 as for Cue 1), and the interaction of the two terms. We included nuisance regressors to establish that the results were not independent of the direction and latency of the saccade to the mask, the prior trial reward, lick rate, and viewing duration (Methods, Eq. 4). The analysis showed that post-revealing responses were common in significant fractions of cells and were dominated by the uncertainty and interaction effects. The uncertainty coefficients reached the largest magnitude at ~600 ms after revealing Cue 2 and were negative, showing that the dip after a Cue 2 signaling no-reward was larger if Cue 2 resolved uncertainty both on average (Fig. 6a, bottom, yellow) and for many individual cells (Fig. 6a, top colormap; note the darker hues around 600 ms). The interaction coefficients were positive, showing that uncertainty enhanced the positive effect of reward on average (Fig. 6a, bottom, pink) and in many individual cells (Fig. 6a, bottom colormap).

Given that the neurons encode the resolution of uncertainty through visual cues, we asked if they also anticipate information delivered by the outcome itself. However, examining the free-viewing epoch responses on no-reveal trials, when the monkeys expected uncertainty to be resolved by the outcome itself, produced no evidence of an anticipatory response (Fig. 6b). This finding, together with a lack of response to reward delivery, suggested that the LIP neurons’ sensitivity to reward and uncertainty was specific to contexts in which the reward information was delivered visually.

Control analyses ruled out alternative interpretations of the reveal-related responses. We found no correlations between the reward and uncertainty coefficients in the pre- and post-saccadic responses (and conducted the analyses after subtracting pre-reveal firing rates to remove the preceding effects of Cue 1), ruling out the post-reveal responses were mere continuations of the neurons’ earlier reward and uncertainty sensitivity. Second, the reveal-related responses peaked at ~600 ms, long after Cue 2 was covered again by the mask, ruling out a relation with viewing duration (additionally, VD was included as a regressor-of-no-interest; *Methods*, Eq. 4). Finally, analysis of free-viewing saccades ruled out an explanation in terms of saccade motor plans. Counting the fraction of free-viewing saccades with vectors within 45° of each cell’s preferred direction showed that the monkeys made more saccades in the neurons’ preferred direction when Cue 2 announced a lack of reward (both in the entire epoch; 41.9 ± 1% on no-reward trials vs 32.6 ± 1% on reward trials; *p* < 0.05, *n* = 68 sessions; and when we measured smaller time bins spanning the free-viewing epoch). This is opposite to a motor hypothesis, which predicts that the monkeys would make fewer RF-directed saccades on trials with a lower post-revealing response. Thus, the post-reveal responses reflected a bona fide nonspatial response to the information conveyed by Cue 2.

## Discussion

The control of attention and gaze is traditionally explained in a reinforcement learning framework, based on the utility of these cognitive processes in increasing instrumental rewards—i.e., enhancing the probability of success of incentivized actions^1,23,24^. However, computational studies show that the mechanisms controlling spontaneous, noninstrumental attention differ substantially from those driven by instrumental incentives^1,3,9,12,23^. Intrinsically motivated information demand has widespread behavioral consequences for curiosity^23^, decision making, and personality traits^11^, highlighting the importance of understanding its cellular mechanisms.

Here we show that cortical responses of visual priority are independently enhanced by two variables that govern noninstrumental information demand: the valence that a stimulus is expected to have and the uncertainty that the stimulus is expected to resolve. The two factors explain distinct portions of firing rate variability and have different behavioral correlations. We discuss the implications of these findings for the mechanisms of information gathering in attentional and executive networks.

LIP neurons have long been proposed to encode the relative importance (priority) of competing for visual stimuli for guiding saccades and attention. The neurons are known to integrate bottom-up and top-down factors that determine the attentional weight and do so independently of motor modality—i.e., whether a stimulus guides a bar press or instructs a saccade toward or away from the stimulus^25–28^. Thus, the visuo-spatial selectivity shown by the cells has been interpreted as encoding the priority of competing for visual stimuli in an effector-general fashion and informing downstream mechanisms that compute decisions about whether and how to act on the information^14,25,28,29^.

While abundant evidence supports this idea, little is known about how priority is computed. Previous studies have focused exclusively on the LIP neurons’ sensitivity to instrumental rewards^4,21,30^, but our results show that the neurons are also sensitive to noninstrumental conditions. We showed that the cells are independently modulated by noninstrumental valence and uncertainty and, consistent with the integrative nature of the priority map, these modulations converge on a common population of cells. Moreover, consistent with the visual rather than motor nature of the neurons’ responses, the uncertainty modulations correlated with the monkeys’ behavioral sensitivity over the course of a session but independently of immediate saccade plans. Notably, the neurons did not signal informativeness per se, since they responded similarly to Cue 1 and the mask, while the informativeness of the stimuli was inversely related (Fig. 1b). Thus, they encoded a prospective response—the priority of gathering additional information from a visual stimulus given the valence and uncertainty resolution it is expected to have.

We also show that, in addition to their pre-saccadic responses, the neurons had post-saccadic responses to the obtained information. In trials in which the monkeys revealed Cue 2, the neurons encoded if the cue signaled a reward or a lack of reward and the uncertainty that this signal resolved, long after the RF had moved away from Cue 2. This suggests that LIP receives nonspatial feedback about the obtained information and may integrate the selection of informative stimuli with the information obtained from the stimuli.

The valence and uncertainty responses in LIP cells had complex relationships with the monkeys’ behavior, suggesting that their contributions to information gathering depend crucially on additional structures. While our understanding of these structures is incomplete, the available evidence suggests that they include areas involved in value and executive function.

According to the expected value of control theory, cognitive processes like attention, learning, and memory are controlled by a network of executive functions, which includes the dACC and neuromodulatory systems and monitors the rewards of a task and the effort that one should invest in a task^31^. A recent neurocomputational model postulates that the dACC monitors reward rates by virtue of inputs from midbrain dopamine cells and, upon detecting a “need for control”, regulates behavioral output by enhancing the appropriate cognitive function^32^.

The model was shown to account for adaptive, context-dependent regulation of memory, learning rates, and physical effort^32^ and we recently showed that it also explains the uncertainty-related enhancement in LIP cells^23^. A recent report shows that a network including the dACC detects noninstrumental uncertainty in a task similar to our own^18^. These cells may produce visual modulations in posterior areas by promoting the release of neuromodulators like norepinephrine, dopamine, or acetylcholine that enhance sensory gain^23^ (see also refs. 17,33,34) or through cortical routes via the dorsolateral prefrontal cortex and frontal eye field^35–37^.

The existing literature has primarily focused on the behavioral effects of uncertainty that are global and generalized, like heightened arousal, increased learning rates, higher behavioral stochasticity, and pupil size modulations (e.g., refs. 36,38). In contrast, the effects we report involve the prioritization of specific visual stimuli and most likely arise from interactions between uncertainty signals that are not spatially tuned and spatially-specific visual representations^23^. In our task, uncertainty and rewards enhanced firing rates both for visual stimuli and at non-stimulated locations, but we speculate that this is due to our use of displays with isolated visual stimuli. Thus, uncertainty may produce a global increase in visual gain, which, in the presence of competing distractors, would strengthen winner-take-all selection dynamics and enhance the selectivity for informative stimuli^39^.

Our comparisons of behavior and neural activity highlight the interactions between valence-driven and uncertainty-driven information demand and suggest that these interactions are individually variable and involve mechanisms that extend beyond the parietal lobe.

Computational and behavioral studies suggest that reward and uncertainty have complex interactive roles in information gathering. On the one hand, the information that animals can access is practically infinite, and subjective utility is crucial for constraining the stimuli to which animals devote their resources. Thus, when behavior is motivated by instrumental rewards, animals appropriately focus on items that are relevant to obtaining those rewards^36,40^. Similarly, when behavior is intrinsically motivated, the anticipatory utility can focus an individual’s inquiries on topics that resonate with their preferred outcomes and longer-term goals.

On the other hand, rewards can spuriously interfere with information gathering. People are distracted by reward-associated stimuli that are irrelevant to a task^41^ and inefficiently demand information from stimuli that have higher rewards but resolve less uncertainty^12^.

This complex relationship implies that the efficient control of attention would ideally allow individuals to flexibly use or filter out reward drives depending on the behavioral context. This may explain findings that humans show marked individual variability in their sensitivity to anticipatory utility and the ability to decouple it from uncertainty-driven information demand^12,11^. Our present results show that analogous variability is present in monkeys. MK1 was relatively more sensitive to uncertainty, and MK2 was relatively more sensitive to rewards. Moreover, MK1, but not MK2, showed a negative correlation between valence and uncertainty sensitivity, consistent with an ability to dissociate the two motives.

Strikingly, these individual differences were not encoded in LIP cells, suggesting that they depend on mechanisms that are largely independent of the parietal cortex. We speculate that valence and uncertainty modulations can arise through multiple circuits, and the circuits that involve LIP may be more or less aligned with those modulating the behavioral output. Our finding that uncertainty modulations in LIP cells correlate with behavior suggests that the uncertainty signals conveyed to LIP overlap with those that determine final decisions about information gathering. This view is consistent with prior results implicating the human parietal cortex in unbiased visual discrimination^42^ and probabilistic reasoning^43,44^. In contrast, our finding that reward modulations in LIP cells do not consistently correlate with behavior suggests that the valence response modulating LIP can, at least in some individuals, differ from that modulating behavior. This proposal resonates with a recent conclusion by Charpentier and colleagues that, in humans, uncertainty- and valence-driven information demand are associated with, respectively, the orbitofrontal cortex versus the mesolimbic reward circuitry^7^. Thus, uncertainty-based information gathering may be driven primarily by circuits that are closely related to cortical areas—including the orbitofrontal and parietal cortex—while the impact of valence depends more strongly on subcortical reward mechanisms.

In sum, our results demonstrate the utility of investigating the cellular mechanisms of noninstrumental information and the insights these investigations can provide about attentional and executive mechanisms in humans and monkeys.

## Methods

### General

Data were collected from two adult male rhesus monkeys using standard behavioral and neurophysiological techniques^45^. All methods were approved by the Animal Care and Use Committees of Columbia University and the New York State Psychiatric Institute as complying with the guidelines within the Public Health Service Guide for the Care and Use of Laboratory Animals. Behavioral control was implemented in MonkeyLogic, stimuli were presented on a Mitsubishi Diamond Pro 2070 monitor (30.4 × 40.6 cm viewing area), eye tracking was performed using an Applied Science Laboratories model 5000 (digitized at 240 Hz), licking was recorded with an in-house device that detected interruptions in a laser beam produced by extensions of the monkeys’ tongue, and action potentials were recorded using an APM digital processing module (Fred Haer). Individual electrodes (glass-coated tungsten electrodes, Alpha Omega, the impedance at 1 kHz: 0.5–1MΩ) were inserted in daily sessions and aimed at the lateral bank of the intraparietal sulcus based on stereotactic coordinates and structural magnetic resonance imaging. Data analysis was performed with MATLAB (R2020b) library functions and custom scripts.

### Memory-guided saccade task

After obtaining a well-isolated waveform, a neuron was first screened with a standard MGS task in which a peripheral target was flashed for 300 ms while the monkeys maintained central fixation, and, after a 500 ms delay period, the monkeys were rewarded for making a saccade to the remembered target location. Neurons were further tested only if they had spatially tuned visual and delay period responses on this task (Supplementary Fig. 2). For these cells, the RF was mapped by conducting the MGS at the same locations used in the information-seeking task, including the estimated RF center and two equally eccentric locations spaced at 120° intervals.

### Information seeking task

The monkey fixated on a central point to initiate a trial. A pattern (Cue 1) indicating 0, 50, or 100% reward probability then appeared for 400 ms, followed by a 1000 ms delay period and the onset of a white mask concealing Cue 2. The locations of Cue 1 and Cue 2 were randomly selected from 3 possible equi-eccentric and equidistant locations, with the constraint that they did not overlap in a trial. The fixation point was removed simultaneously with mask onset, and the monkeys were free to deploy gaze for 2500 ms. For the first 1500 ms of this epoch, the mask remained visible, and the monkeys could reveal Cue 2 by fixating the mask for a minimum of 200 ms (ensuring that their gaze did not spuriously land on the mask). If revealed, Cue 2 was visible for 300 ms and was again concealed by the mask regardless of the monkey’s gaze location. After the 1500 ms epoch, the mask disappeared, and, after a 1000 ms blank screen, the trial ended with a tone and the delivery of the outcome—reward or no-reward—as predicted by the cues regardless of free-viewing behavior. All temporal intervals between Cue 1 onset and outcome were fixed, removing uncertainty about the delay to reward delivery.

The cues were square, colored checkerboard (“Mondrians”) measuring 3 deg of visual angle that were equated for luminance and discriminability^46^. Three patterns were consistently associated with 0% reward probability, two with 50% probability, and three with 100% reward probability. The monkeys were first extensively familiarized with the 8 cue patterns using a passive version of the task in which they fixated centrally while a pattern was presented for 400 ms in the periphery, followed by delivery of the appropriate outcome after a 1 s delay. Familiarization continued until the monkeys showed reliable discrimination of Cue 1 probability in their anticipatory licking response. At this stage, the monkeys were presented with an information-seeking task. During the information-seeking task, each trial was first randomly assigned a reward probability and outcome (reward/no-reward). Then, one of the patterns signaling the appropriate probability was randomly assigned as Cue 1, with each Cue 1 pattern followed by two equiprobable Cue 2 patterns signaling the appropriate outcome. Anticipatory licking was monitored throughout, and each information-seeking session started with a few “reminder” trials of the passive task, ensuring that the monkeys had a good grasp of the cue-reward contingencies (Supplementary Fig. 1).

### Data analysis

During neural recordings, one of the three possible locations was in the RF of the cell, while the others were outside the RF. We analyzed data from completed trials in which the monkeys successfully maintained fixation and either did not reveal Cue 2 or did so within 600 ms of mask onset (on average, 607 trials per cell; for simplicity, we excluded <0.5% of trials in which Cue 2 was revealed after 600 ms or later).

### Behavior

Saccade onset and offsets were detected based on velocity and acceleration criteria using custom-made software^47^. To analyze information-seeking behavior, we fit each session’s data into a linear model

1 $${{{{{\bf{R}}}}}}{{{{{\bf{E}}}}}}{{{{{\bf{V}}}}}}={\beta }_{0}{{{\bf{1}}}}\;+\;{\beta }_{{{{{{\mathrm{REW}}}}}}\_{{{{{\mathrm{reveal}}}}}}}{{{{{\bf{R}}}}}}{{{{{\bf{E}}}}}}{{{{{\bf{W}}}}}}\;+\;{\beta }_{{{{{{\mathrm{UNC}}}}}}\_{{{{{\mathrm{reveal}}}}}}}{{{{{\bf{U}}}}}}{{{{{\bf{N}}}}}}{{{{{\bf{C}}}}}}$$

where **REV** contains 1 for trials where Cue 2 was revealed and 0 otherwise, **REW** is the reward probability signaled by Cue 1 (0, 0.5, or 1), and **UNC** is the associated uncertainty (0, 1, 0).

To analyze viewing duration (**VD**), we fit the reveal trials in each session to a linear model:

2 $${{{{{\bf{V}}}}}}{{{{{\bf{D}}}}}}={\beta }_{0}{{{\bf{1}}}}\;+\;{\beta }_{{{{{{\mathrm{REW2}}}}}}\_{{{{{\mathrm{VD}}}}}}}{{{{{\bf{R}}}}}}{{{{{\bf{E}}}}}}{{{{{\bf{W}}}}}}{{{\bf2}}}\;+\;{\beta }_{{{{{{\mathrm{UNC}}}}}}\_{{{{{\mathrm{VD}}}}}}}{{{{{\bf{U}}}}}}{{{{{\bf{N}}}}}}{{{{{\bf{C}}}}}}\,+\,{\beta }_{{{{{{\mathrm{REW2}}}}}}\ast {{{{{\mathrm{UNC}}}}}}\_{{{{{\mathrm{VD}}}}}}}{{{{{\bf{R}}}}}}{{{{{\bf{E}}}}}}{{{{{\bf{W}}}}}}{{{\bf2}}} * {{{{{\bf{U}}}}}}{{{{{\bf{N}}}}}}{{{{{\bf{C}}}}}}$$

where **VD** is the time from removal of the mask and eye’s exit from a 2 deg window surrounding the mask, **REW2** is the reward probability signaled by Cue 2 (0 or 1), **UNC** is the uncertainty resolved by Cue 2 defined as above, and ∗ denotes element-wise multiplication.

### Neural analysis

We computed z-scored firing rates (**FRz**) by convolving each trial’s spike trains with a Gaussian filter (sigma = 30 ms) and z-scoring within a cell using all the time points and trials collected from that cell. We analyzed each cell using statistical models as noted below, and report coefficient distributions over all the cells that had at least 2 trials for each condition required to estimate the model regressor.

To extract the time-resolved effects of reward and uncertainty in the information-seeking task (Fig. 2), we fit **FRz** with 1 ms resolution throughout the period of interest using the equation:

3 $${{{{{\bf{{{F}}}}}}}}{{{{{{\bf{{{R}}}}}}}}}_{zt}=	 \;{\beta }_{0}{{{{{\bf{1}}}}}}\;+\;{\beta }_{{{{{{\mathrm{REW}}}}}}\_{{{{{\mathrm{reveal}}}}}}}\,{{{{{\bf{{{REW}}}}}}}}\;+\;{\beta }_{{{{{{\mathrm{UNC}}}}}}\_{{{{{\mathrm{reveal}}}}}}}{{{{{\bf{{{UNC}}}}}}}}\;+\;{\beta }_{{{{{{\mathrm{DIR}}}}}}}{{{{{\bf{{{DIR}}}}}}}}\\ 	+ {\beta }_{{{{{{\mathrm{LAT}}}}}}}{{{{{\bf{{{LAT}}}}}}}}\;+\;{\beta }_{{{{{{\mathrm{PR}}}}}}}{{{{{\bf{{{PR}}}}}}}}\;+\;{\beta }_{{{{{{\mathrm{LIC}}}}}}{K}_{t}}{{{{{\bf{{{LIC}}}}}}}}{{{{{{\bf{{{K}}}}}}}}}_{{{{{{\boldsymbol{t}}}}}}}$$

where **FRz**_**t**_ is the z-scored firing rate at time *t*, **REW** and **UNC** are defined as in Eq. 1, **DIR** is the direction of the first free-viewing saccade (1 if directed in a ±45° cone centered on the RF; 0 otherwise), **LAT** is the latency of the first free-viewing saccade, **PR** is the reward outcome on the preceding trial (1 if rewarded, 0 otherwise), **LICK**_**t**_ is the binary licking status at time t (1 if licking, 0 otherwise).

Consistent with previous findings^48^, we found that, when Cue 1 appeared inside the RF, 50% of the cells showed sensitivity to the visual pattern independently of reward probability. However, pattern-selective and non-selective cells did not consistently differ in their reward and uncertainty coefficients (*p* > 0.05 for both *β*_*UNC_neural*_, and *β*_*REW_neural*_), confirming that reward and uncertainty exerted independent effects.

In the post-reveal analysis (Fig. 6), in order to control for effects of reward and uncertainty merely based on Cue 1, we first subtracted the mean over the 100 ms before Cue 2 onset from the z-scored firing rate on each trial. We then used these mean-subtracted firing rates (**FRzd**_**t**_) to estimate the effects produced specifically by Cue 2 by fitting the equation:

4 $${{{{{\bf{{{F}}}}}}}}{{{{{{\bf{{{R}}}}}}}}}_{{{{{{\boldsymbol{zdt}}}}}}}=	 \;{\beta }_{0}{{{{{\bf{1}}}}}}\;+\;{\beta }_{{{{{{\mathrm{UNC}}}}}}\_{{{{{\mathrm{neural}}}}}}}{{{{{\bf{{{UNC}}}}}}}}\;+\;{\beta }_{{{{{{\mathrm{REW2}}}}}}\_{{{{{\mathrm{neural}}}}}}}{{{{{\bf{{{REW}}}}}}}}{{{{{\bf{2}}}}}} \\ 	+{\beta }_{{{{{{\mathrm{UNC}}}}}}\ast {{{{{\mathrm{REW2}}}}}}\_{{{{{\mathrm{neural}}}}}}}{{{{{\bf{{{UNC}}}}}}}} * {{{{{\bf{{{REW}}}}}}}}{{{{{\bf{2}}}}}}\;+\;{\beta }_{{{{{{\mathrm{DIR}}}}}}}{{{{{\bf{{{DIR}}}}}}}}\;+\;{\beta }_{{{{{{\mathrm{LAT}}}}}}}{{{{{\bf{{{LAT}}}}}}}}\\ 	+{\beta }_{{{{{{\mathrm{PR}}}}}}}{{{{{\bf{{{PR}}}}}}}}+{\beta }_{{{{{{\mathrm{VD}}}}}}}{{{{{\bf{{{VD}}}}}}}}\;+\;{\beta }_{{{{{{\mathrm{LIC}}}}}}{{{{{{\mathrm{K}}}}}}}_{t}}{{{{{\bf{{{LIC}}}}}}}}{{{{{{\bf{{{K}}}}}}}}}_{{{{{{\boldsymbol{t}}}}}}}$$

where **DIR**, **LAT**, **PR**, and **LICK**_**t**_ are defined as in Eq. (3) (with **DIR** and **LAT** referring to the first saccade away from fixation, which triggered the reveal), and **VD** defined as in Eq. (2).

Although an ideal design would have allowed for cross-validated estimates of significance, this would require careful a priori computations of the number of trials in training and validation sets. However, our focus on spontaneous rather than instrumental behavior meant that trial numbers for many analyses were under the animals’ rather than the experimenters’ control (e.g., there were very few trials in which the monkeys chose to reveal Cue 2 at 0% reward probability, etc.). Thus, to enhance the reliability of traditional significance tests, we used nonparametric tests and repeated the analyses using both beta coefficients and fraction of variance explained, as described next.

One set of analyses focused on the regression coefficients for reward and uncertainty—*β*_*REW*_ and *β*_*UNC*_ in the various models. Because models that contain only a reward or only an uncertainty regressor are nested into those that contain both regressors, finding a significant beta coefficient in a two-parameter model indicates that the respective term accounts for a significant portion of variability that is not explained by the other term. This inference assumes that the regressors have low correlations, to preclude overfitting or misallocation of variance. Thus, we report the correlation among the reward and uncertainty regressors for each analysis in the text (below 0.1 in most individual sessions/cells).

We verified these results using additional R^2^ analysis estimating the variance that was uniquely explained by each variable. We defined variance that was uniquely explained by a regressor as the R^2^ of the full model minus the R^2^ a partial model that omitted the regressor, i.e., the increase in R^2^ by including the target regressor. To determine the significance of the excess R^2^ we conducted a 1-sided permutation test and determined if the observed excess R^2^ was larger than the 95% chance excess R^2^ obtained from randomly shuffled data.

### Reporting summary

Further information on research design is available in the Nature Research Reporting Summary linked to this article.

## Supplementary information

Supplementary Information

Reporting Summary

## Data Availability

The data that support the findings of this study are available in figshare with the identifier 10.6084/m9.figshare.20666349. Source data are provided with this paper.

## Source data

Source Data
