# Supplementary material for: Uncertainty modulates visual maps during noninstrumental information demand
Source: Nat Commun. 2022 Oct 7;13:5911. doi: 10.1038/s41467-022-33585-2 (PMC9547007; doi:10.1038/s41467-022-33585-2)
Supplement: Supplementary file 1 — Supplementary Information [file 41467_2022_33585_MOESM1_ESM.pdf]

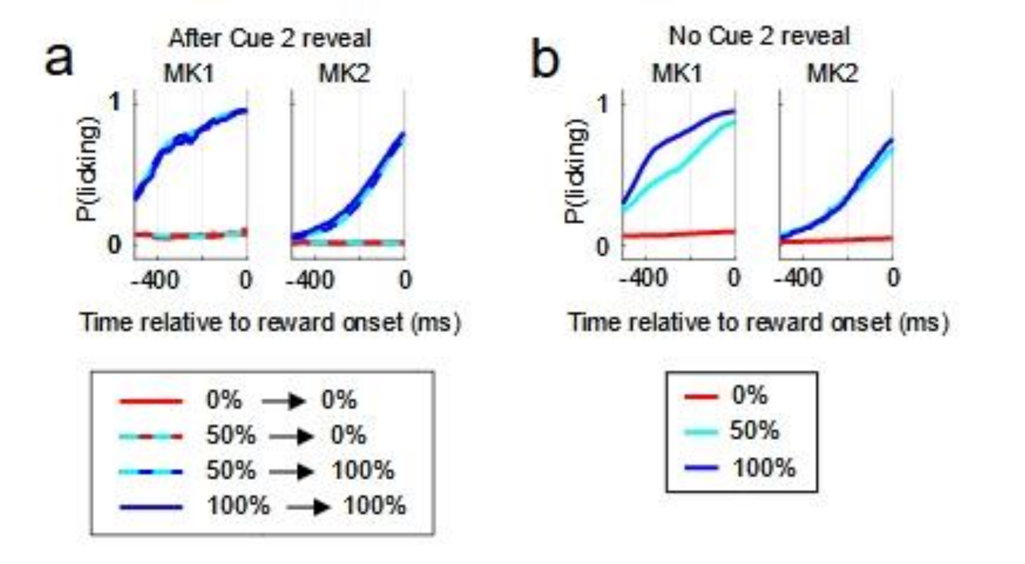

Supplementary Figure 1

Anticipatory licking behavior shows that the monkeys were familiar with and attended to the cued reward probabilities. a. Probability of licking before reward onset after the monkeys revealed Cue 2. Both monkeys licked if Cue 2 signaled a reward but not if it signaled a lack of reward. **b.** Probability of licking on no-reveal trials scales with the probability signaled by Cue 1, showing that the monkeys expected rewards even if they did not reveal Cue 2 (1-way ANOVA, $p < 10^{-43}$). The analysis was conducted on the subset of cells with sufficient no-reveal trials at each reward probability (n = 37 in MK1, n = 31 in MK2) and was significant in each monkey.

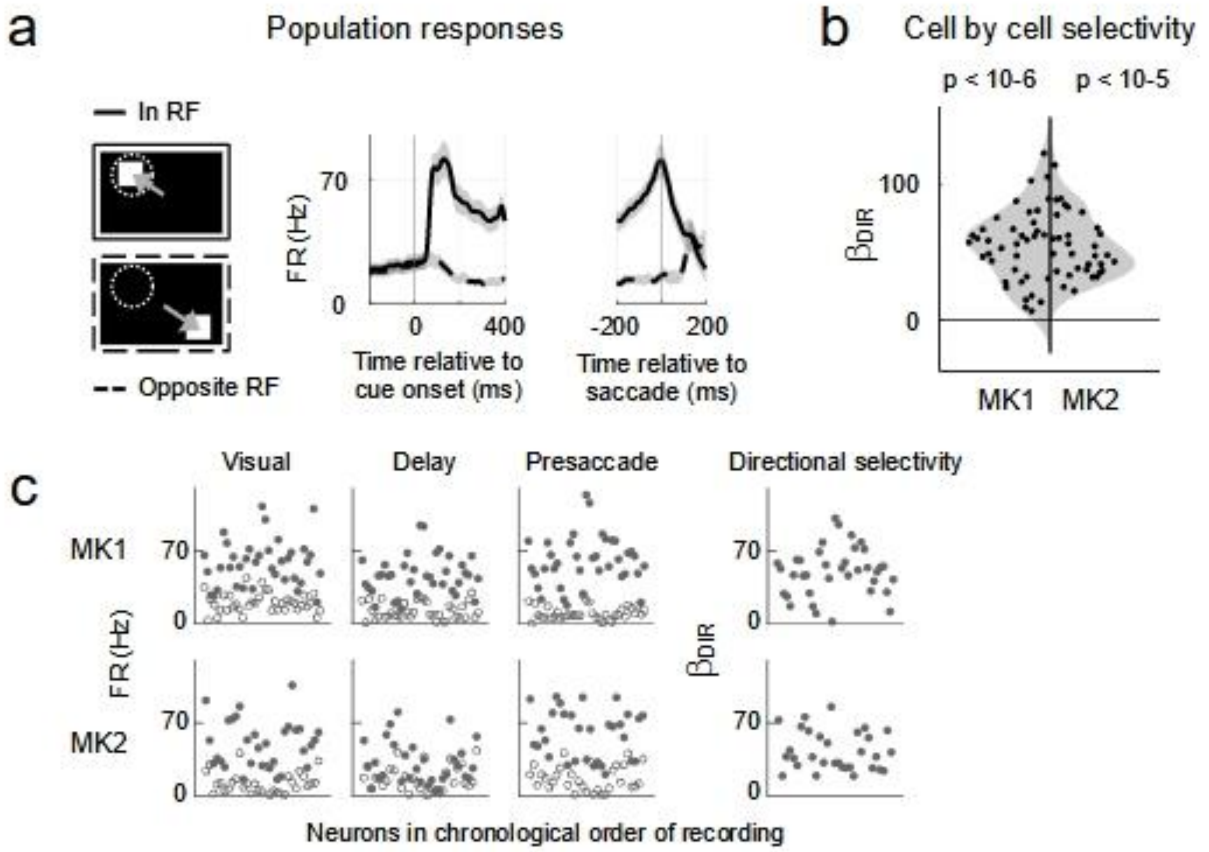

Supplementary Figure 2

The recorded cells were spatially selective and the population is stationary. a. PSTHs showing firing rates in the memory-guided saccade task, for target location/saccade goals inside the RF (solid) and opposite the RF (dashed; mean and 2 SEM, n = 68 cells). **b.** Regression coefficients measuring saccade selectivity in the 100 ms before saccade were computed by fitting $\mathbf{FR} = \beta_0 * \mathbf{1} + \beta_{DIR} * \mathbf{DIR}$, where $\mathbf{DIR}$ = 1 (0) if the saccade goal was inside (opposite) RF). Each point shows the β_{DIR} coefficient for one cell. The distributions (shading) were well above zero (signed-rank test against 0, MK1: $p < 10^{-6}$, n = 37; MK2: $p < 10^{-5}$, n = 31), and coefficients were individually significant in all but one cell in MK1 (which only showed spatially tuned visual but not pre-saccadic activity). **c.** Average firing rates (first 3 panels) and directional selectivity (last panel) in the memory-guided saccade task as a function of recording sessions in chronological order. In the first 3 panels, filled and open points show trials in which the target/saccade goals were, respectively, inside and opposite the RF. No measure showed significant time trends, indicating that the population of sampled neurons was consistent across the experiment.

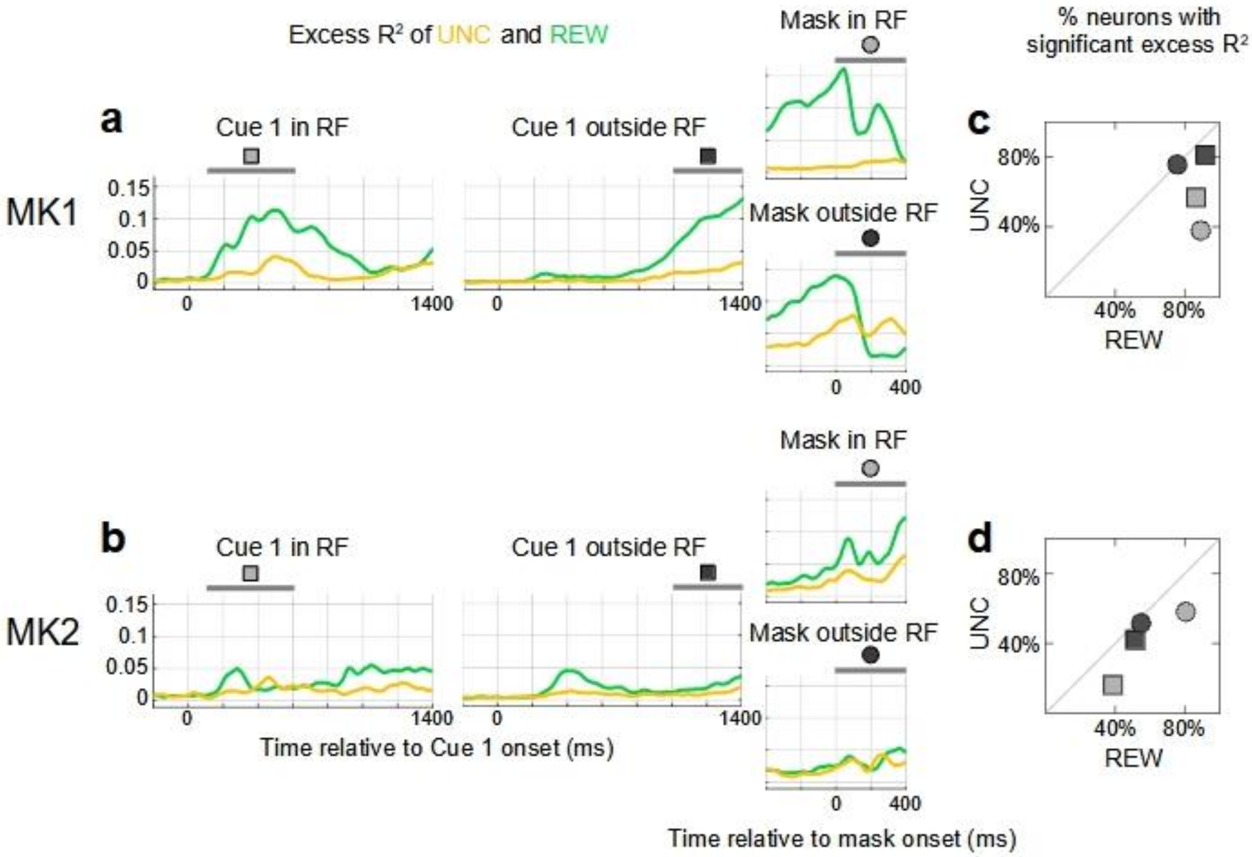

Supplementary Figure 3
Analysis of the unique variance of LIP activity that is explained by uncertainty and reward.

a and b. Time courses of the unique variance that is explained in MK1 (**a**) and MK2 (**b**) corresponding to the coefficients in **Fig. 2**. Each panel shows the excess R^2 (above a shuffled control; see *Methods*) that is uniquely explained by reward (green) and uncertainty (yellow), computed in a time-resolved fashion and averaged across cells using the same methods as in **Fig. 2**. The symbols and gray bars above the panels indicate the symbols and time windows used for the summary statistics in panels c and d. **c and d.** Percent of neurons with significant ($p < 0.05$) unique variance explained by uncertainty (ordinate) and reward (abscissa) of MK1 (c) and MK2 (d). Light square: 100-600ms after Cue 1 onset in RF; dark square: 1000-1400ms after Cue 1 onset outside RF; light circle: 0-400ms after mask onset in RF; dark circle: 0-400ms after mask onset outside RF (and following Cue 1 outside RF). For reference, the fraction of unique variance explained by saccade direction was smaller and was significant in only 9.5% of neurons for the Cue 1 response and 27% of neurons for the mask response (ranges across stimulus locations and monkeys; respectively, 6%-16% and 14% to 51%).

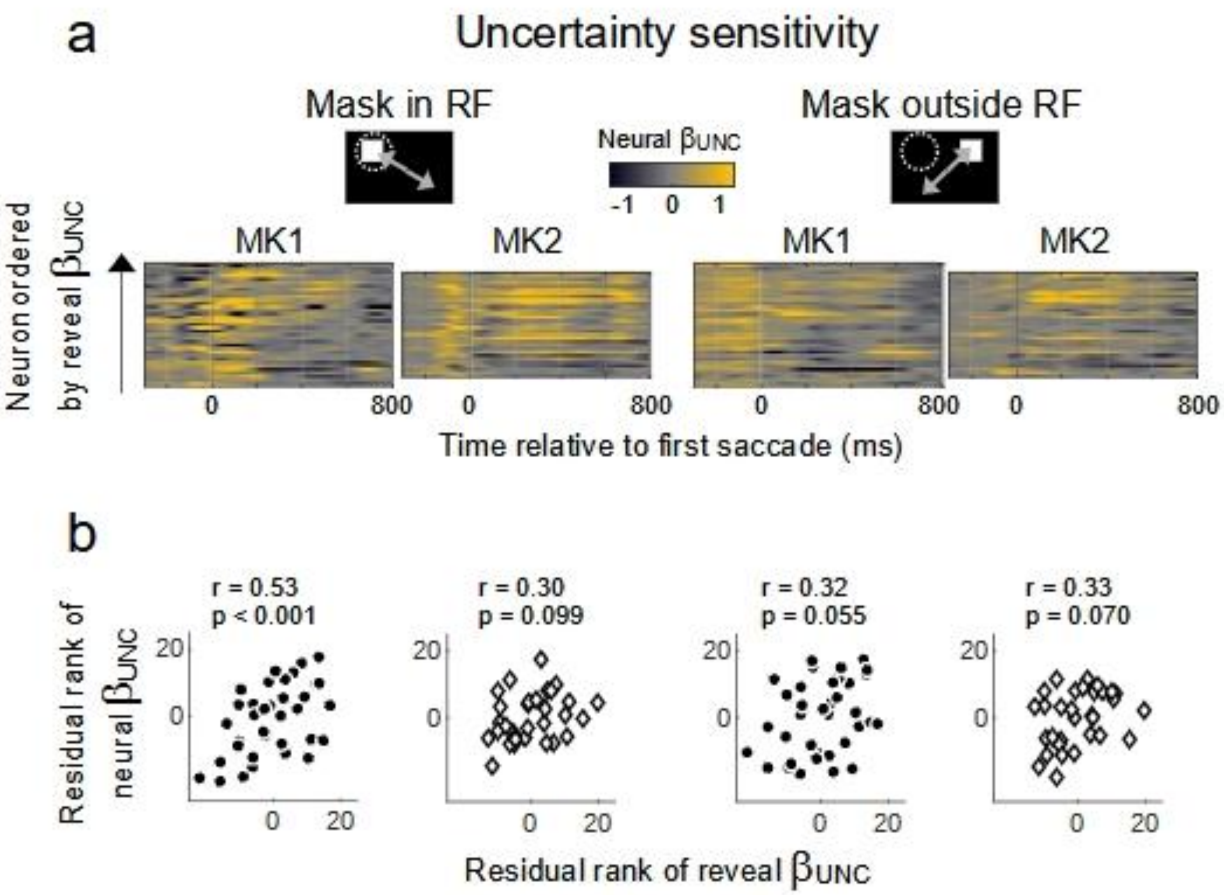

Supplementary Figure 4
Neural-behavioral correlations remain consistent in all trials. a. Correlations between neural and behavioral β_{UNC} were generally replicated when we included both reveal and no-reveal trials. **b.** Correlations of residual rank obtained after controlling for effects on viewing duration (**VD**) with both reveal and no-reveal trials included. R and p-values are of Pearson correlation analyses. Note that, when including reveal trials, post-saccadic neural responses encode the revealed information (cf **Fig. 6**), adding noise that accounts for the marginally significant effects in MK2.

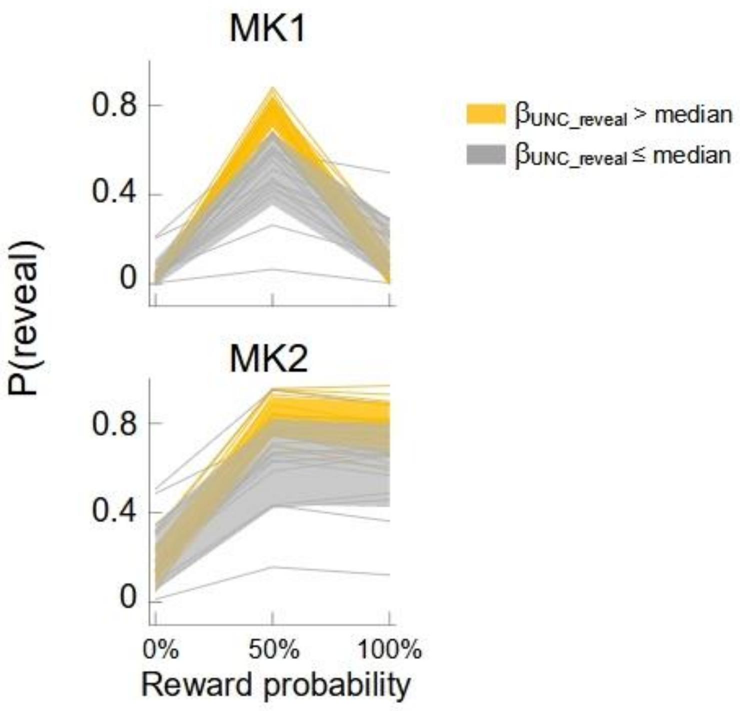

Supplementary Figure 5
Uncertainty and reward behavior drivers are negatively related in MK1 and positively related in MK2. Each line is one session and shading represents ±1 standard deviation around the mean. The sessions are color coded according to a median split of the β_{UNC_reveal} coefficients. For MK1, sessions with high β_{UNC_reveal} have high reveal rate at 50% and low reveal rate at 100%, accounting for the negative correlation between β_{UNC_reveal} and β_{REW_reveal} in **Fig. 5a**, top. For MK2, sessions with high β_{UNC_reveal} have high reveal rates at both 50% and 100%, explaining the positive correlation in **Fig. 5a**, bottom.
