# Supplementary material for: Uncertainty modulates visual maps during noninstrumental information demand
Source: Nat Commun. 2022 Oct 7;13:5911. doi: 10.1038/s41467-022-33585-2 (PMC9547007; doi:10.1038/s41467-022-33585-2)
Supplement: Supplementary file 2 — Reporting Summary [file 41467_2022_33585_MOESM2_ESM.pdf]

nature portfolio

Corresponding author(s): Jacqueline Gottlieb

Last updated by author(s): Aug 26, 2022

Reporting Summary

Nature Portfolio wishes to improve the reproducibility of the work that we publish. This form provides structure for consistency and transparency in reporting. For further information on Nature Portfolio policies, see our Editorial Policies and the Editorial Policy Checklist.

Statistics

For all statistical analyses, confirm that the following items are present in the figure legend, table legend, main text, or Methods section.

n/a	Confirmed	
☐	☒	The exact sample size (n) for each experimental group/condition, given as a discrete number and unit of measurement
☐	☒	A statement on whether measurements were taken from distinct samples or whether the same sample was measured repeatedly
☐	☒	The statistical test(s) used AND whether they are one- or two-sided<br>*Only common tests should be described solely by name; describe more complex techniques in the Methods section.*
☐	☒	A description of all covariates tested
☐	☒	A description of any assumptions or corrections, such as tests of normality and adjustment for multiple comparisons
☐	☒	A full description of the statistical parameters including central tendency (e.g. means) or other basic estimates (e.g. regression coefficient) AND variation (e.g. standard deviation) or associated estimates of uncertainty (e.g. confidence intervals)
☐	☒	For null hypothesis testing, the test statistic (e.g. F, t, r) with confidence intervals, effect sizes, degrees of freedom and P value noted<br>*Give P values as exact values whenever suitable.*
☒	☐	For Bayesian analysis, information on the choice of priors and Markov chain Monte Carlo settings
☒	☐	For hierarchical and complex designs, identification of the appropriate level for tests and full reporting of outcomes
☐	☒	Estimates of effect sizes (e.g. Cohen's d, Pearson's r), indicating how they were calculated

Our web collection on statistics for biologists contains articles on many of the points above.

Software and code

Policy information about availability of computer code

Data collection	MonkeyLogic Version 1
Data analysis	MATLAB R2020b library functions in custom scripts

For manuscripts utilizing custom algorithms or software that are central to the research but not yet described in published literature, software must be made available to editors and reviewers. We strongly encourage code deposition in a community repository (e.g. GitHub). See the Nature Portfolio guidelines for submitting code & software for further information.

Data

Policy information about availability of data

All manuscripts must include a data availability statement. This statement should provide the following information, where applicable:

- Accession codes, unique identifiers, or web links for publicly available datasets
- A description of any restrictions on data availability
- For clinical datasets or third party data, please ensure that the statement adheres to our policy

The data that support the findings of this study are available in figshare with the identifier 10.6084/m9.figshare.20666349.

Field-specific reporting

For a reference copy of the document with all sections, see nature.com/documents/nr-reporting-summary-flat.pdf

Life sciences study design

All studies must disclose on these points even when the disclosure is negative.

Sample size	Two animal subjects with a total of 68 neurons. Based on the literature [4], this is a common sample size for the effects we expected in LIP. No statistical methods were used to pre-determine sample sizes.
Data exclusions	Neurons were selected based on pre-defined, standard, electrophysiological response patterns, as outlined in manuscript.<br>Error trials were not considered in analysis of neural responses. As described in manuscript for each analysis, neurons with insufficient trials were excluded.
Replication	Results were replicated in two animals and in 68 independently sampled cells.
Randomization	The process of selecting the neurons is a random sample of LIP on a given experimental session.<br>All experimental conditions were represented with equal proportion in random order within each session.
Blinding	Investigators were not blinded. Because all conditions are presented in each experiment session, data pre-processing procedures were the same regardless of conditions, and results are based on statistical analyses, blinding is irrelevant.

Reporting for specific materials, systems and methods

We require information from authors about some types of materials, experimental systems and methods used in many studies. Here, indicate whether each material, system or method listed is relevant to your study. If you are not sure if a list item applies to your research, read the appropriate section before selecting a response.

Materials & experimental systems

n/a	Involved in the study	
☒	☐	Antibodies
☒	☐	Eukaryotic cell lines
☒	☐	Palaeontology and archaeology
☐	☒	Animals and other organisms
☒	☐	Human research participants
☒	☐	Clinical data
☒	☐	Dual use research of concern

Methods

n/a	Involved in the study	
☒	☐	ChIP-seq
☒	☐	Flow cytometry
☒	☐	MRI-based neuroimaging

Animals and other organisms

Policy information about studies involving animals; ARRIVE guidelines recommended for reporting animal research

Laboratory animals	2 Rhesus monkeys (macaca mulatta), male, adult, age 15
Wild animals	Wild animals were not used in this study.
Field-collected samples	Field samples were not used in this study.
Ethics oversight	All methods were approved by the Animal Care and Use Committees of Columbia University and New York State Psychiatric Institute as complying with the guidelines within the Public Health Service Guide for the Care and Use of Laboratory Animals.

Note that full information on the approval of the study protocol must also be provided in the manuscript.

March 2021
